# Unmapped RNA Virus Diversity in Termites and Their Symbionts

**DOI:** 10.3390/v12101145

**Published:** 2020-10-09

**Authors:** Callum Le Lay, Mang Shi, Aleš Buček, Thomas Bourguignon, Nathan Lo, Edward C. Holmes

**Affiliations:** 1 Marie Bashir Institute for Infectious Diseases and Biosecurity, The University of Sydney, Sydney 2006, New South Wales, Australia; callum.lelay@sydney.edu.au (C.L.L.); mang.shi@sydney.edu.au (M.S.); 2 School of Life and Environmental Sciences, The University of Sydney, Sydney 2006, New South Wales, Australia; nathan.lo@sydney.edu.au; 3 School of Medical Sciences, The University of Sydney, Sydney 2006, New South Wales, Australia; 4 Okinawa Institute of Science and Technology Graduate University, Tancha, Onna-son, Okinawa 904-0495, Japan; bucek.ales@gmail.com (A.B.); thomas.bourguignon@oist.jp (T.B.); 5 Faculty of Tropical AgriSciences, Czech University of Life Sciences, 165 00 Prague, Czech Republic

**Keywords:** RNA viruses, evolution, ecology, metatranscriptomics, RNA sequencing, termites

## Abstract

Despite their ecological importance, nothing is known about the diversity and abundance of RNA viruses in termites (Termitoidae). We used a metatranscriptomics approach to determine the RNA virome structure of 50 diverse species of termite that differ in both phylogenetic position and colony composition. From these samples, we identified 67 novel RNA viruses, characterized their genomes, quantified their abundance and inferred their evolutionary history. These viruses were found within or similar to those from the *Togaviridae, Iflaviridae, Polycipiviridae, Flaviviridae, Leviviridae, Narnaviridae, Mitoviridae, Lispivirdae, Phasmaviridae*, *Picobirnaviridae* and *Partitiviridae*. However, all viruses identified were novel and divergent, exhibiting only 20% to 45% amino acid identity to previously identified viruses. Our analysis suggested that 17 of the viruses identified were termite-infecting, with the remainder likely associated with the termite microbiome or diet. Unclassified sobemo-like and bunya-like viruses dominated termite viromes, while most of the phylogenetic diversity was provided by the picobirna- and mitovirus-like viruses. Of note was the identification of a novel flavi-like virus most closely related to those found in marine vertebrates and invertebrates. Notably, the sampling procedure had the strongest association with virome composition, with greater RNA virome diversity in libraries prepared from whole termite bodies than those that only sampled heads.

## 1. Introduction

Invertebrates, particularly insects, are the most diverse lineage of animals [1]. This diversity is likely to be paralleled by the diversity of their viromes, rendering invertebrates a potentially rich resource of novel viruses. The metatranscriptomic (i.e., total RNA sequencing) study of a variety of invertebrate taxa has revealed that these organisms harbor an enormous diversity of RNA viruses, currently accounting for ~36% of the complete RNA virus genomes in the RefSeq database [2]. Not only are invertebrate RNA viruses diverse, but the viromes of individual species are often complex with levels of abundance that are often far higher than those seen in vertebrate species [3,4]. Despite this metagenomic revolution, the current sample of the virosphere remains fragmentary, leading to substantial gaps in our understanding of RNA virus diversity, evolution and ecology. Evidently, a simple first step in changing this picture is to characterize more of those RNA viruses present in invertebrates, which in turn will help to establish a new framework for studying virus ecology and evolution.

One group of invertebrate organisms of particular interest are termites (Termitoidae, order Blattodea). These hugely important animals can be divided into two groups: (i) the paraphyletic lower termites that are wood-feeding and largely rely on gut protists (Parabasalia and Oxymonadida) [5,6] to metabolize their diet and (ii) the monophyletic higher termites that lost protozoa, entirely rely on gut bacteria and archaea to metabolize their diet and include species that can feed on soil, humus, grass or wood [6,7]. There are more than 3000 currently known species of termites, and they are found on all continents except Antarctica, although most abundantly in warm/tropical areas [5]. Regardless of termite group, each worker in a colony harbors a complex bacterial community [6,8]. These features, along with high host densities and group living, make it reasonable to expect high levels of virus diversity and transmission in termite populations, and that their RNA virome will contain a variety of plant, bacterial, fungal and protist-associated viruses alongside those that infect termites themselves [9,10,11]. For example, bees are similarly social insects with a range of associations to microorganisms, and metatranscriptomic studies have revealed that they harbor more complex viromes than most insects [12,13,14,15].

To date, however, remarkably little is known about termite viruses, with only a small number of DNA viruses identified: entomopoxvirus [16,17] and nuclear polyhedrosis virus [18] have been identified in *Reticulitermes flavipes* and *Kalotermes flavicollis*, Caudovirales bacteriophage were found infecting termite symbionts [8,19,20], and single-stranded DNA viruses from the families *Circoviridae*, *Microviridae*, and *Genomviridae* have been detected in several termite species [8,21]. In marked contrast, there has been no research into the RNA virome of termites. At the time of writing, the closest taxa examined are cockroaches (from the same order, Blattodea), in which 4 viruses were identified in one study [4] and 12 from another [22]. These viruses fall within the *Narnaviridae*, *Phasmaviridae*, *Xinmoviridae*, *Phenuiviridae*, *Rhabdoviridae*, *Orthomyxoviridae*, and chuviruses, along with a single orthomyxo-like virus and a single nairo-related virus. Members of the *Parvoviridae* (ssDNA) have also been found in cockroaches [23,24].

The central aim of this study was to come to a better understanding of the diversity and abundance of RNA viruses associated with termites. As well as documenting virus diversity, we addressed whether the diversity of RNA viruses reflects termite phylogeny and/or host ecology. To this end, we performed a metatranscriptomic analysis of a range of termite species that represent key points in the termite phylogeny.

## 2. Materials and Methods

### 2.1. Termite Collection, RNA Isolation and Sequencing

Australian samples, of which there were 10, were collected from the termite species *Occasitermes* sp., *Coptotermes acinaciformis*, *Heterotermes ferox* and *Schedorhinotermes intermedius* colonies at Manly Vale (New South Wales, Australia) in August 2016. Similarly, samples from *Cryptotermes secundus* and *Mastotermes darwiniensis* colonies were collected from Darwin (Northern Territory, Australia) in May 2016. Samples comprised more than five individual termites from an individual colony and were immediately stored at −80 °C. Two samples were collected for each of *Occasitermes* sp., *H. ferox* and *S. intermedius*, representing two separate colonies. Members of *M. darwiniensis* were collected individually and later pooled to produce two libraries: library 19, comprising worker caste termites, and library 20 that consists of soldier caste termites.

All of these Australian termites (i.e., libraries 3–4, 11–15, 18–20) were subject to a “whole-body” procedure in which they were homogenized using a TissueRuptor (QIAGEN, Hilden, Germany), and RNA was extracted using the RNeasy Plus Mini Kit (QIAGEN, Hilden, Germany). RNA was depleted of ribosomal (r) RNA using the Ribo-Zero Gold rRNA Removal Kit (Epidemiology) (Illumina, San Diego, CA, USA) and cDNA libraries were prepared with the TruSeq Total RNA Library Prep kit (Illumina, San Diego, CA, USA). Libraries were sequenced on an Illumina HiSeq 2500 platform (100 bp paired-end reads) (Illumina, San Diego, CA, USA). The Sequence Read Archive (SRA) accession numbers for these libraries are: SRR8924822-SRR8924831.

The second set of termites comprised 44 samples, of which 10 produced libraries containing credible RNA viruses (libraries 1, 2, 5–10, 16–17). These were collected as described in Bucek et al. [7] from various geographic locations (Supplementary Table S1) and are designated as “head-only” libraries. Again, termites were collected and stored at −80 °C until RNA extraction. Rather than utilizing whole body tissue samples, the heads of 2–15 termites were removed and RNA extracted using a standard phenol–chloroform procedure with TRIzol reagent (Life Technologies, Grand Island, NY, USA). RNA samples were poly(A)+ enriched and cDNA libraries were sequenced using an Illumina HiSeq 2500 platform (125 bp paired-end reads) (Illumina, San Diego, CA, USA). The SRA accession numbers for these libraries are: SRR12736817, SRR9968561-SRR9968586, SRR9968593-SRR9968607, SRR9968609, and SRR9968615 (Supplementary Table S1).

### 2.2. Assembly and Virus Identification

Sequenced reads were trimmed using Trimmomatic and de novo assembled using Trinity (2.5.1) [25]. BLASTn (2.6.0) and diamond BLASTx (0.9.10) [26] searches on contigs were used to identify contigs that hit to viruses. These putative contigs were then manually parsed to identify viruses. Only contigs with credible, significant BLAST hits (e-value < 1 × 10^−5^) and without non-viral CD-search [27] hits were retained. GeneMark.hmm PROKARYOTIC v3.26 [28] was used to predict virus open reading frames (ORFs), the amino acid sequences of which were then extracted. To estimate virus abundance in each library, the Expectation-Maximization (RSEM; 1.3.0) [29] tool was used to determine the number of transcripts per million (TPM) for all contigs. Detailed descriptions of the final set of sequences with sufficient identity to viruses that we refer to here as “viruses”, including sequenced genome length, abundance and virus group assignment, are provided in Supplementary Table S2. The consensus sequences of all novel viruses identified here have been submitted to GenBank and assigned accession numbers MW052060- MW052147 and are also described in Supplementary Table S2.

### 2.3. Virus Abundance Measurements

The abundance level of each virus was estimated as the percentage of viral reads from the total read count. Abundances greater than 0.01% were arbitrarily considered as representing a “high” virus abundance, values between 0.01% and 0.001% were considered as “moderate” abundance, while those less than 0.001% were considered as “low” abundance. To reduce the impact of false-positives due to index-hopping, we assumed that viruses were the result of contamination from another library if the total read count was less than 0.1% of the highest count for that virus among the other libraries. This led to the removal of one putative Pafsystermes virus (326 reads in library 20 compared to ~180,000 in libraries 14 and 15).

### 2.4. Viruses Excluded from the Analysis

A number of potential virus sequences were excluded: contaminating RNA viruses, likely endogenous RNA viruses and all hits to DNA viruses. Contaminating RNA viruses were bovine viral diarrhea virus 1 and human picobirnavirus. Sequences with significant BLAST hits but lacking a complete genome in high abundance and containing indels that interrupt the ORF predictions were considered to be endogenous and excluded. In particular, almost all samples contained several sequences with similarity to viruses in the Mono–Chu group of RNA viruses that appeared to be endogenous: Hubei chuvirus, Hubei chuvirus 4, Hubei oodonate virus 11, Hubei rhabdo-like virus 3, Lampyris noctiluca chuvirus-like virus 1, Wuchan romanomermis nematode virus 3 and Hubei orthoptera virus 5. The majority of these sequences had no significant full length (or close to full length) hits to the National Center for Biotechnology Information (NCBI) conserved domain database (CDD) and/or no gene-sized ORFs. Because of the difficulty in distinguishing DNA viruses from host genes, particularly those with double-stranded genomes, these were similarly excluded.

### 2.5. Phylogenetic Analysis

All viral ORFs containing sequences corresponding to the virus RNA-dependent RNA polymerase (RdRp) were used to infer maximum likelihood phylogenetic trees based on their amino acid sequences. For ease of analysis, prior to the sequence alignment, RdRp amino acid sequences were placed into the following groups defined previously [4]: the *Flaviviridae*, Hepe–Virga, Bunya–Arena, Mono–Chu, Luteo–Sobemo, Picorna–Calici, Narna–Levi and Partiti–Picobirna groups. Multiple sequence alignments were constructed using MAFFT (v7.402) [30] with 1000 iterative refinements. Alignments were then trimmed using TrimAL (v1.4.1) [31] to remove poorly aligned sequence positions. Two phylogenetic trees were then estimated for each group of viruses: (i) An initial phylogeny to place the termite viruses in their overall context was inferred encompassing the entire virus group as described above, and (ii) a second more specific tree was made for all clades of termite viruses and their closest relatives. Viruses with replicase sequences sharing >95% amino acid similarity were considered to be members of the same species.

Phylogenetic trees were estimated using the maximum likelihood method available in PhyML [32] employing the Le-Gascuel (LG) model of amino acid substitution, Subtree-Pruning-Regrafting (SPR) tree topology searching, with Shimodaira–Hasegawa (SH)-like approximate likelihood ratio test (aLRT) branch supports used to assess node robustness. The R packages “Analyses of Phylogenetics and Evolution” (APE; v5.3) [33], ggtree (v1.16.6) [34] and phytools (v0.6.99) [35] were used for tree manipulation and final figure production.

### 2.6. Statistical Analysis of Abundance and Diversity

The R packages Vegan (v2.5-6) [36] and Phyloseq (1.30.0) [37] were used to calculate alpha- and beta-diversity statistics for these viromes. The multcomp (v1.4-13) [38] package was used for statistical tests, and ggplot2 (v3.3.0) [39] was used for data visualization. Analysis of variance (using a Chi-square test) of generalized linear models was used to check for significant relationships between termite taxonomy, sampling procedure, termite diet and source country and a variety of ecological measures (total virus abundance and virome diversity and richness).

### 2.7. Statistical Analysis of Abundance and Diversity

Virus names were chosen to provide meaningful information on the host sample in each case, while simultaneously being both succinct and distinctive as recommended by the International Committee on Taxonomy of Viruses (ICTV). Accordingly, all virus names were constructed with a unique, random prefix combined with the “-systermes” suffix—“-sys-”, from Greek, meaning “with”, and “-termes”, from Latin, meaning “woodworm” and the root of the word termite. We therefore use “-systermes” to convey that the named virus was obtained from within termites and/or the community of organisms associated with termites.

## 3. Results

### 3.1. Identification and Annotation of RNA Viruses

We collated samples from 50 species of termite, each of which contained 2 to 25 individual termites. From these samples, we constructed 54 RNA sequencing libraries, with most species represented by a single library (Supplementary Table S1). Libraries produced via the head-only procedure enabled a better association of viruses with the termite host, although they generated fewer data. Across both procedures, the 54 samples yielded an average of 42 million and 34 million sequencing paired-reads, which we reconstructed into an average of 497,862 and 166,012 contigs, for the whole-body and head-only libraries, respectively.

### 3.2. RNA Virus Diversity in Termites

**Figure 1:**
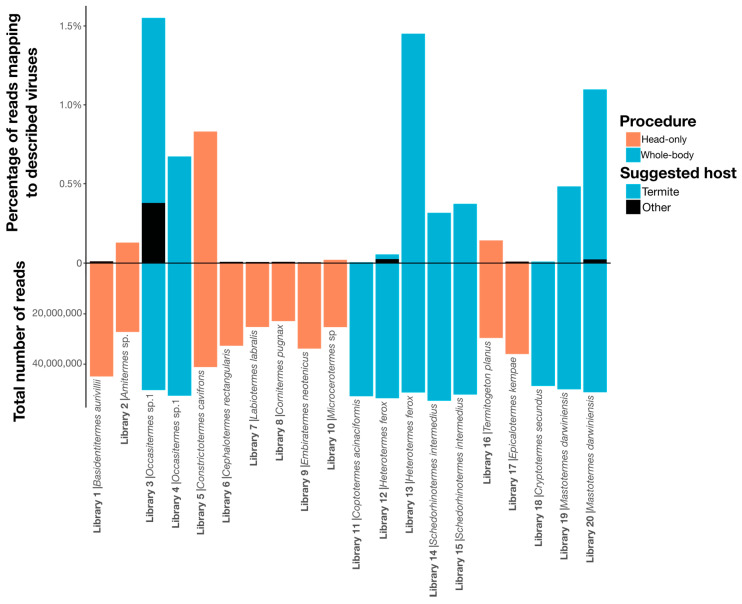
Bar graph depicting the number of reads in each sequencing library, below the x-axis, and the percentage of these reads that map to viruses described in this study, above the x-axis. Each library is labeled by name and termite species sampled. Bars are colored by sampling procedure. Sequenced contigs were annotated using BLAST (BLASTx and BLASTn) and conserved domain-based searches (CD-search) to identify putative viral contigs, excluding those matching commonly contaminating RNA viruses, endogenous viruses, retroviruses and any hits to DNA viruses (see Methods). Overall, an average of 68% of contigs per sample was unidentified (no significant BLAST hit), and 27.4% hit host sequences (i.e., from the phylum Arthropoda), 1.3% hit bacterial sequences and 0.024% of all contigs had a significant hit to a virus genome. After predicting open reading frames (ORFs) for contigs determined to represent true virus genomes, CD-search hits were used for annotation, and the predicted replicase genes were incorporated into phylogenetic trees to determine their identity.

**Figure 2:**
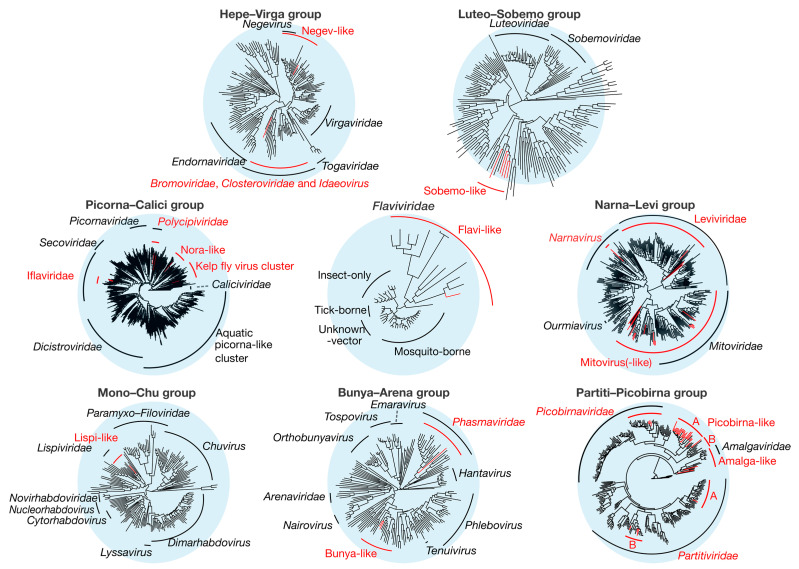
Overall phylogenetic trees representing each of the nine groups of RNA viruses (Table 2). Phylogenies were inferred using the gene encoding the RdRp, with the virus taxa (usually genera) identified in this study shown in red. Taxonomic groups of interest are labeled on the trees for reference. The sequence alignments used to infer the individual phylogenies are described in Supplementary Table S3. All trees are unrooted with branch length scaled to the number of amino acid substitutions per site.

**Figure 3:**
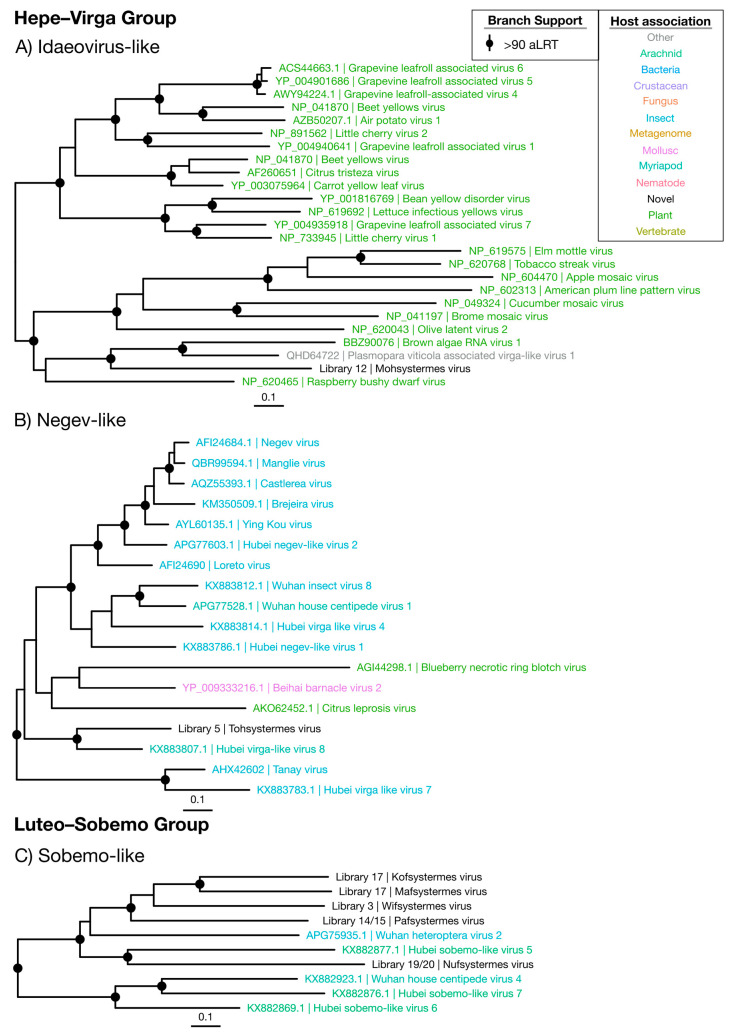
Phylogenetic trees of the viruses newly discovered here and their relatives in the Hepe–Virga and Luteo–Sobemo groups. Newly identified viruses are labeled with black text in the format “<Library #|Virus name>”, while related viruses are colored by their host association and are labeled in the format “<Replicase protein accession>|<Virus name>”. Black dots at tree nodes indicate high branch support (aLRT > 90).

**Figure 4:**
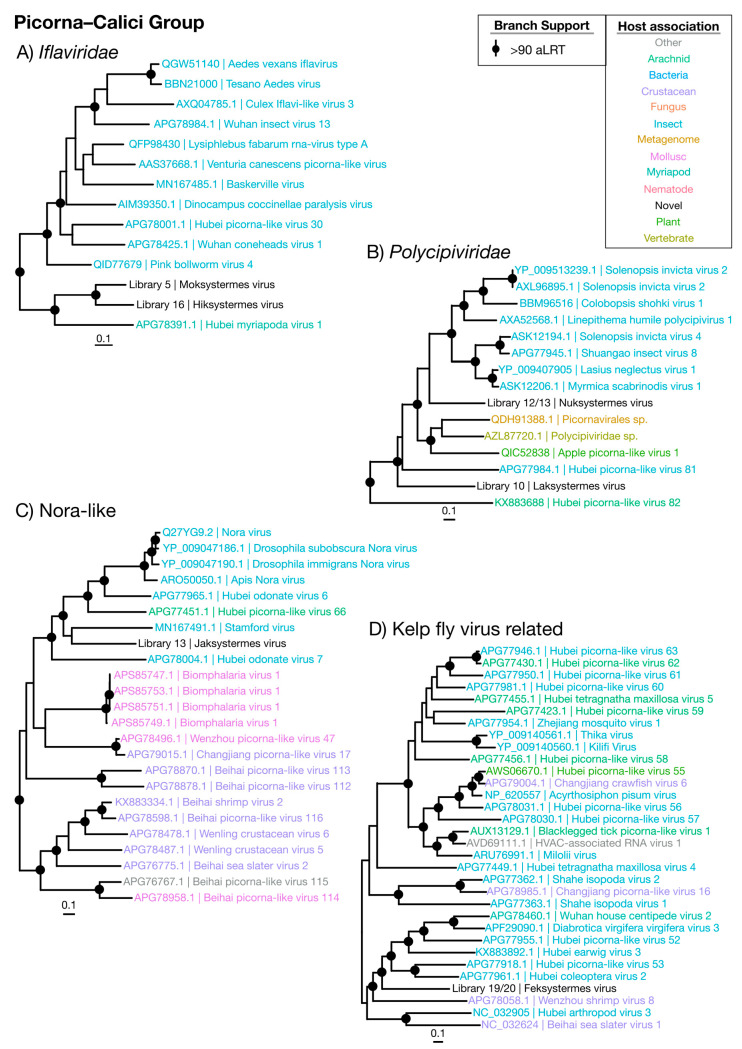
Phylogenetic trees of the viruses newly discovered here and their relatives in the Picorna–Calici group. Newly identified viruses are labeled with black text in the format “<Library #|Virus name>”, while related viruses are colored by their host association and are labeled in the format “<Replicase protein accession>|<Virus name>”. Black dots at tree nodes indicate high branch support (aLRT > 90).

**Figure 5:**
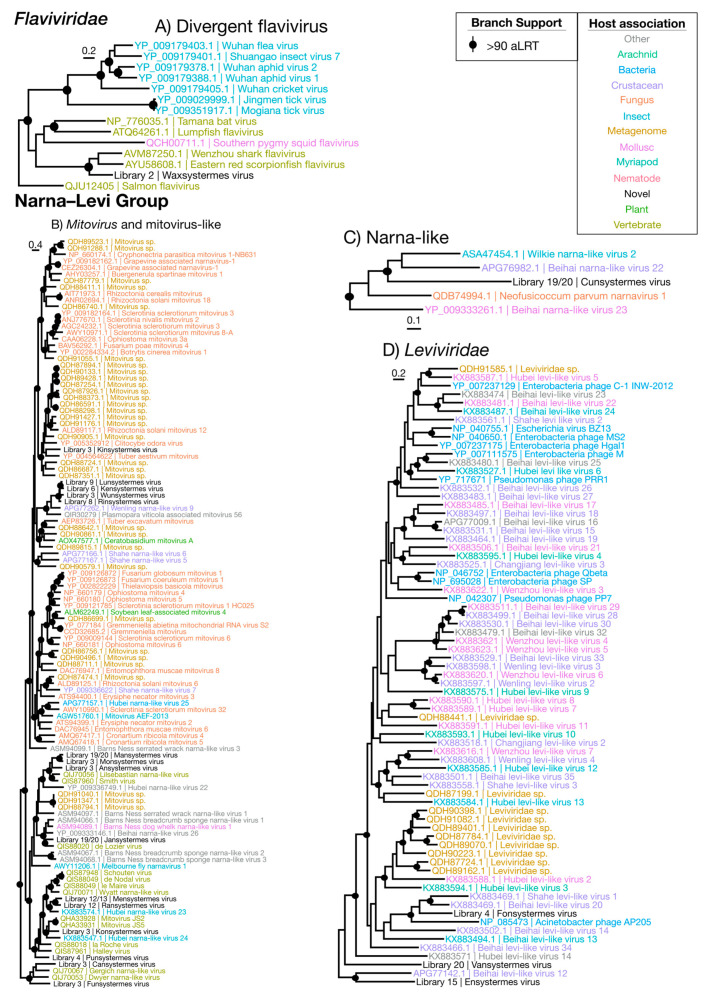
Phylogenetic trees of the viruses newly discovered here and their relatives in the *Flaviviridae* and the Narna–Levi group. Newly identified viruses are labeled with black text in the format “<Library #|Virus name>”, while related viruses are colored by their host association and are labeled in the format “<Replicase protein accession>|<Virus name>”. Black dots at tree nodes indicate high branch support (aLRT > 90).

**Figure 6:**
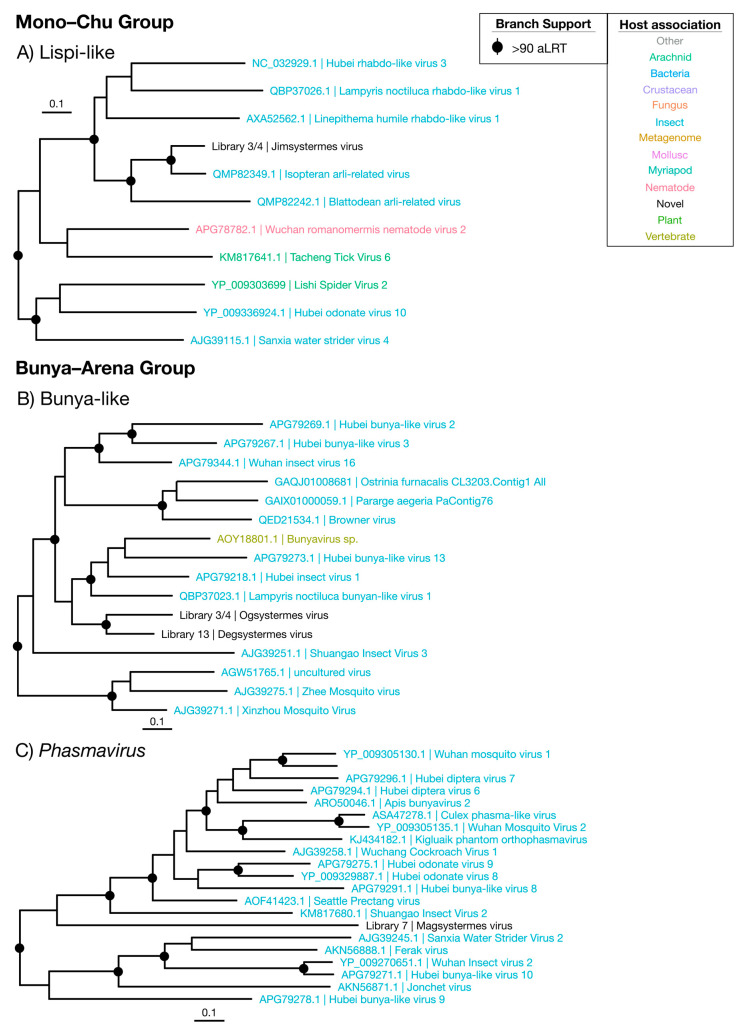
Phylogenetic trees of the viruses newly discovered here and their relatives in the Mono–Chu and Bunya–Arena groups. Newly identified viruses are labeled with black text in the format “<Library #|Virus name>”, while related viruses are colored by their host association and are labeled in the format “<Replicase protein accession>|<Virus name>”. Black dots at tree nodes indicate high branch support (aLRT > 90).

**Figure 7:**
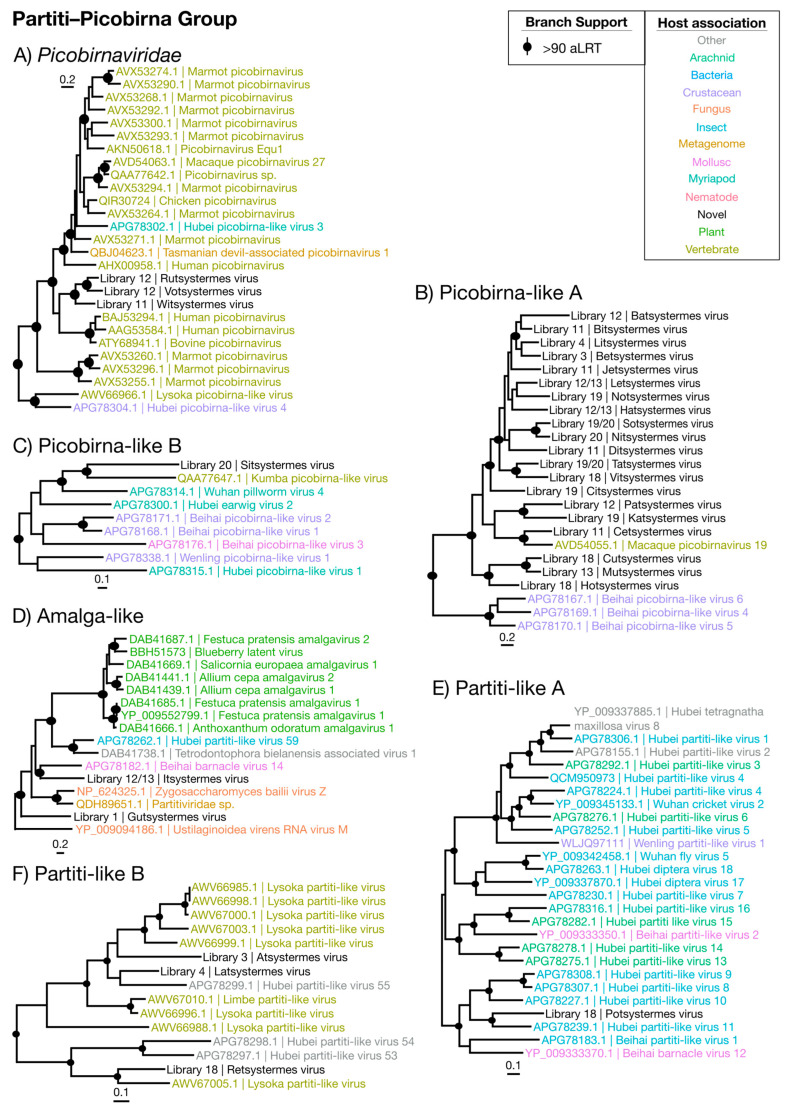
Phylogenetic trees of the viruses newly discovered here and their relatives in the Partiti–Picobirna group. Newly identified viruses are labeled with black text in the format “<Library #|Virus name>”, while related viruses are colored by their host association and are labeled in the format “<Replicase protein accession>|<Virus name>”. Black dots at tree nodes indicate high branch support (aLRT > 90).

Overall, we identified 67 novel viruses (i.e., novel sequences with virus identity) from 20 sequencing libraries (Figure 1, Table 1 and full details in Supplementary Table S2), which could be placed into 9 phylogenetic groups (Figure 2). As many viruses had close relatives to taxonomically unassigned viruses determined by Shi et al. [4], we used similar groupings of virus families to aid identification. In total, 33 positive-sense single-stranded (ss) RNA virus genomes were identified and assigned to six groups based on phylogenetic analysis: the Hepe–Virga, Luteo–Sobemo, Picorna–Calici, Narna–Levi and *Flaviviridae* groups [4]. Similarly, four negative-sense ssRNA virus genomes were recovered and assigned to the Mono–Chu and Bunya–Arena groups, while 30 double-stranded (ds) RNA viruses were identified and assigned to the Partiti–Picobirna group. Within these major phylogenetic groups, the 67 viruses identified here were assigned to 21 clades that enabled a more specific classification and interpretation (Figure 3, Figure 4, Figure 5, Figure 6 and Figure 7).

**Figure 8:**
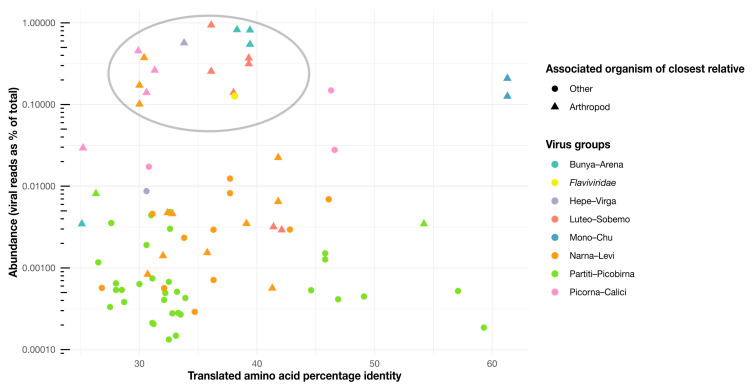
Abundance scatterplot for the novel viruses identified here. Each virus is represented by a single point, with the color indicating the taxonomic group the virus belongs to and the shape indicating the host organism of the closest virus relative (arthropod hosts are shown as triangles). The y-axis depicts the log abundance of the viruses, while the x-axis shows the translated amino acid percentage identity for each virus from their closest relatives. The gray circle indicates a cluster of viruses in relatively high abundance (generalized linear model, *df* = 81, *p* = 2 × 10^−16^) and closest relatives that commonly have arthropod associations (Pearson’s Chi-sq, *df* = 81, *p* = 4.6 × 10^−6^).

We also attempted to infer host–virus relationships (i.e., which of the viruses identified likely infect termites themselves as opposed to elements of their diet and other microorganisms), although these are necessarily tentative. Host assignments were informed by virus abundances and the host associations of related viruses and the procedure used to produce each sequencing library. We assume that viruses at a high abundance are more likely to be infecting the termite host, particularly when these viruses are closely related to arthropod-associated viruses (Figure 8) and found in head-only samples (where the gut, containing possible symbiont hosts, is excluded). We now discuss each of the major groups of viruses in turn.

### 3.3. Positive-Sense ssRNA Viruses

#### 3.3.1. Hepe–Virga Viruses

**Figure 9:**
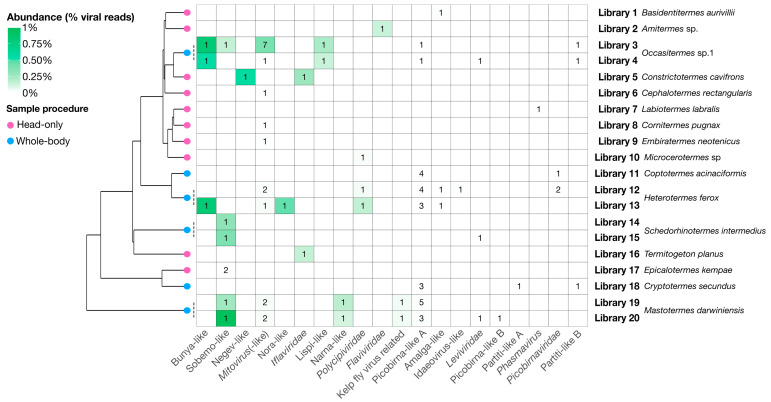
Heatmap showing number of virus genomes and their relative abundance in each library. Virus clades are shown on the x-axis by decreasing total abundance, libraries are given on the right y-axis ordered by the phylogenetic relationships of the termites in each library. The abundance, as a percentage of the total reads, of each genome is presented as a heat map while the number of genomes found for each group is labeled for each cell. The schematic phylogenetic tree on the left is adapted from Bucek et al. [7] and represents phylogenetic relationships between termites associated with each library. Tip nodes on this tree are colored by procedure used to collect termites and process them to libraries: Blue nodes represent the “whole-body” procedure, while pink nodes represent the “head-only” procedure.

Two novel viruses were identified within the Hepe–Virga group (Figure 2 and Figure 3). Of these, the newly defined “Mohsystermes virus” is grouped with members of the *Idaeovirus* genus, a sparsely sampled group of plant-infecting viruses, and found at a moderate abundance of 0.0087% of the total reads in the *H. ferox* library 12 (Figure 3A and Figure 9). Mohsystermes virus is considered to have a close to complete genome, as it has a similar total genome size (9888 nt) to its relatives Brown algae RNA virus 1 (BAV1), *Plasmopara viticola* associated virga-like virus 1 (PVV) and Raspberry bushy dwarf virus. Similar to BAV1 and PVV, Mohsystermes virus is lacking a significant hit to a methyl-transferase (Supplementary Figure S1). As Mohsystermes virus has a phylogenetic background with an association with plant hosts and is found at a moderate abundance, we infer the host for this virus to be a constituent of the termite diet.

Tohsystermes virus is the second virus identified in this group and has a far greater certainty with respect to both its host association and genome completeness. This virus has a 10,376 nt genome with predicted domains similar to its closest relative, the Myriapod-associated Hubei virga-like virus 8, which has a 10,433 nt genome and 33.8% amino acid identity with Tohsystermes virus (Figure 3B, Supplementary Figure S1). As Tohsystermes virus falls within the insect-infecting Negev-like virus group that contains insect-associated viruses, has a very high abundance (0.54%) and was obtained from the head-only *Constrictotermes cavifrons* library, we suggest that it is likely a truly termite-infecting virus.

#### 3.3.2. Luteo–Sobemo Viruses

The Luteo–Sobemo group comprises the plant-infecting *Luteoviridae* and *Sobemoviridae* families, although the majority of viruses in this group are formally unassigned (Figure 2). Indeed, it is striking that the five Luteo–Sobemo viruses identified in this study—Pafsystermes virus, Kofsystermes virus, Mafsystermes virus, Nufsystermes virus and Wifsystermes virus—form a clade that is markedly divergent from either family: These viruses were placed with a set of unassigned arthropod-associated viruses found by Shi et al. [4] (Figure 3C).

Almost all sobemo-like virus genomes identified are close to complete with a 2500 nt to 3000 nt segment, containing a RdRp domain (CDD: cl02808) and a smaller second segment, with a viral coat domain (CDD: cl29941) (Supplementary Figure S2). Notably, the ORF structure predicted for Wifystermes virus requires the use of a variant genetic code in which UGA is translated as tryptophan. This alternative codon is used in the yeast and mitochondrial genetic codes, both of which can be used to predict ORFs in the other four sobemo-like viruses.

The five novel viruses identified here often dominate the viromes in which they are detected, with abundance levels greater than 0.003% and as high as 0.94% (Figure 9). Interestingly, Nufsystermes virus and Pafsystermes virus were each detected in the paired libraries from *M. darwiniensis* and *S. intermedius*, respectively. Given the very high abundance of these viruses and their phylogenetic relationship to arthropod-associated viruses (Figure 2), we suggest that the novel viruses in this group infect the termite hosts themselves and specifically may be mitochondria-infecting based on their variant genetic code.

#### 3.3.3. Picorna–Calici Viruses

The Picorna–Calici group consists of the large and diverse *Picornaviridae* and the vertebrate-infecting *Caliciviridae*, as well as a wide range of other classified and unclassified viruses. We identified seven novel viruses (Moksystermes virus, Hiksystermes virus, Jaksystermes virus, Nuksystermes virus, Laksystermes virus and Feksystermes virus) that fall within the Picorna–Calici group, although these viruses occupied a range of phylogenetic positions and included some of the most divergent ones found in this study (Figure 1 and Figure 8). More precisely, the viruses identified here fall within the insect-associated *Iflaviridae* (Figure 4A) and *Polycipiviridae* families within the Picorna–Calici group (Figure 4B), as well as in clades of unassigned viruses (Figure 4C,D). Strikingly, all viruses are found in high abundance (0.017% to 0.45%) (Figure 9), and the majority of the viruses related to the Picorna–Caliciviruses found are associated with arthropods. Hence, we suggest that all of these viruses are termite-infecting.

The majority of the Picorna–Calici group identified here are close to complete based on the length of their genomes in comparison to their documented relatives, although they often lack significant hits to known protein domains (Supplementary Figure S3). Jaksystermes virus is ~6000 nt longer than its closest relative, the flea-associated Stamford virus [40]. In addition, Jaksystermes virus contains an unusual hit to HAM1, CDD: cd00515. Nuksystermes virus and Laksystermes virus genomes have a structure which is consistent with that of the *Polycipiviridae*, with a cluster of four ORFs separated from the replicase ORF [41].

#### 3.3.4. Flaviviridae

The *Flaviviridae* are notable in that they infect both invertebrates and vertebrates. Some flaviviruses, such as those from the genus *Flavivirus*, are commonly pathogenic to vertebrates and transmitted among them by arthropod vectors (Figure 2). We identified a single novel member of the *Flaviviridae*—denoted Waxsystermes virus—that is found in high abundance (0.13%) in a head-only library, and which possesses the genome structure common to members of the genus *Flavivirus* (Supplementary Figure S4). Interestingly, Waxsystermes virus falls within a flavi-like virus clade that contains two marine vertebrate-associated viruses: Eastern red scorpionfish flavivirus (51.2% amino acid identity) and Wenzhou shark flavivirus (38.1% amino acid identity) (Figure 5A). The true host for Waxsystermes virus is therefore difficult to determine, and this part of the phylogeny is characterized by a complex mix of invertebrate and vertebrate-associated viruses. While Waxsystermes virus clearly does not infect a marine vertebrate, its high abundance and adherence to the flavivirus genome structure points toward an insect host. We therefore cautiously suggest that Waxsystermes virus is termite-infecting.

#### 3.3.5. Narna–Levi Viruses

The Narna–Levi viruses were the most ubiquitous group of viruses detected in our libraries, with 19 novel viruses identified (Figure 9). The majority of the novel Narna–Levi viruses were placed within and sister to members of the genus *Mitovirus* that typically infects the mitochondria of fungi and of some plants [42,43] (Figure 5B). All possess small, simplistic, single-segment genomes (Supplementary Figure S5) and were detected in low to moderate abundances ranging from 0.00029% to 0.022%. The exception to this is Wunsystermes virus, present in high abundance (0.37%) in an *Occasitermes* library. In addition, Wunsystermes virus, as well as Kinsystermes virus, seemingly utilize UGA codons as tryptophan encoding for their predicted ORFs. Such a codon assignment is commonly seen in fungal viruses [42], although it is occasionally seen in plant-infecting mitoviruses [43] and is used in the invertebrate mitochondrial genetic code. Given the strong association of viruses within this group with fungi, the seven novel Narna–Levi viruses that fall within the Mitovirus genus are likely fungus-associated.

In contrast, the further eight novel viruses that fall basal to this clade—Mansystermes virus, Monsystermes virus, Ansystermes virus, Jansystermes virus, Mensystermes virus, Ransystermes virus, Konsystermes virus and Punsystermes virus—are related to arthropod and vertebrate-associated relatives. This, coupled with their low abundances in whole-body libraries, means that we can make no clear inference to their host.

Of the remaining four viruses identified in this group, three fell within the *Leviviridae* (Figure 5D): Fonsystermes virus, Vansystermes virus and Ensystermes virus. These viruses have genomes structurally similar to those seen in leviviruses, ranging from 3346 nt to 4931 nt (Supplementary Figure S5). All were present in low abundances (0.00057% to 0.0023%) in whole-body libraries and fell within a well-known bacteria-infecting family in the phylogenetic analysis, suggesting that they most likely associated with bacteria.

Finally, Cunsystermes virus has the largest genome of this group at 6454 nt and was detected in a clade basal to the genus *Narnavirus* (Figure 2 and Figure 5C), although its host is unclear. While Cunsystermes virus is present in high abundance in the whole-body *M. darwiniensis* libraries (0.10% and 0.17%), it is also phylogenetically divergent (sharing only 30% amino acid identity with its closest relative, Wilkie narna-like virus 2) and is placed within an under-sampled clade with mixed host associations. We are therefore unable to make a safe inference for the host of Cunsystermes virus.

### 3.4. Negative-Sense ssRNA Viruses

#### 3.4.1. Mono–Chu Viruses

Sequences with similarity to the Mono–Chu viruses were relatively commonplace in the termite libraries, but all but one appeared to be endogenous: These sequences had disjointed ORFs and incomplete genomes. Jimsystermes virus is the only Mono–Chu group virus detected that has a credible genome structure indicative of an exogenous virus, with a mononega-like RdRp (CDD: pfam00946), capsid (CDD: pfam14318) and methyl-transferase (CDD: cl27811) domains detected in its largest ORF (Supplementary Figure S6). This virus is detected in both *Occasitermes* sp. libraries 3 and 4 in high abundances of 0.2% and 0.13% and falls within the *Lispiviridae* with the other insect-associated viruses (Figure 6A) [44]. Isopteran arli-related virus, from *Coptotermes* sp. [22], is the closest relative to Jimsystermes virus with an amino acid identity of 61.3%. Given the clear insect association of the *Lispiviridae* and the high abundance of Jimsystermes virus, we suggest that this virus is insect-infecting.

#### 3.4.2. Bunya–Arena Viruses

The *Bunyavirales* and the *Arenaviridae*, which together comprise the Bunya–Arena group, are associated with both invertebrates and vertebrates. The three novel viruses we detected from this group—denoted Degsystermes virus, Ogsystermes virus and Magsystermes virus—sit phylogenetically with viruses that have a clear association with arthropods, including mosquitoes, fleas, glow-worms and butterflies (Figure 2, Figure 6B,C). Structurally, all three viruses are similar, with genomes that encode a single, large ORF that contains a *Bunyavirales* RdRp domain (CDD: cl01709). Notably, these viruses have some of the highest abundances detected in this study, ranging from 0.24% to 0.82% (Figure 9). While only single, ~6000 nt segments were detected for most of these novel viruses, a possible second 1974 nt segment in a similar abundance to Degsystermes virus was detected in *H. ferox* library 14 (Supplementary Figure S7). A second such segment has been observed in the genomes of the related Hubei insect virus 1 and Hubei bunya-like virus 13.

According to our phylogenetic analysis, Ogsystermes virus and Degsystermes viruses fall in a clade of unassigned bunya-like viruses (Figure 6B), while Magsystermes virus groups within the *Phasmaviridae*, basal to the newly defined *Orthophasmavirus* genus [44] (Figure 6C). Notably, these novel phasmaviruses are highly divergent, with less than 28% amino acid identity to their closest relatives (Figure 8). Due to their relatively high abundance as the dominant components of the viromes in which they are detected, as well as their clustering with other invertebrate viruses in the phylogenetic analysis, we suggest that these are termite-infecting viruses.

### 3.5. Double-Stranded RNA Viruses

#### Partiti–Picobirna Viruses

By far the most numerous and commonly detected group of viruses identified were members of the Partiti–Picobirna group that comprises members of the *Partitiviridae* and *Picobirnaviridae* (Figure 2)—non-enveloped dsRNA viruses with ~4000 nt genomes. The *Partitiviridae* are known to infect plants, fungi and protozoa and are transmitted intracellularly. The *Picobirnaviridae,* however, are more complex. While they are often associated with vertebrate fecal metagenomes [45], there is evidence that they may be infecting bacterial symbionts of vertebrate hosts [46]. We identified 30 novel virus genomes for this group, spanning nine libraries and placed phylogenetically into six clades (Figure 7 and Table 2). No second segment was detected for any virus identified in this group, and genomes detected were typically ~1900 nt and only encoded an RdRp domain (Supplementary Figure S8). Importantly, while these viruses were detected in the lowest abundances seen in this study (0.0001% to 0.008%), several were typically detected per each library and only within the whole-body libraries (Figure 9).

The majority of the viruses in this group were found within and sister to the *Picobirnaviridae* (Figure 2), falling with related viruses sampled from primate feces (Figure 7A–C) [45,46]. The remaining seven partiti-like viruses fell into three groups: two viruses fell basal to members of the genus *Amalgavirus* (Figure 7D), two viruses grouped with unassigned arthropod-associated viruses [4] (Figure 7E), while three viruses grouped with the fecal-associated Lysoka partiti-like viruses (Figure 7F). With their low abundance, previous association with feces and representation in whole-body libraries, we suggest that these novel viruses infect termite gut symbionts.

### 3.6. Correlates of RNA Virome Diversity and Abundance

Due to the small sample size and the confounding country and procedure variables (i.e., all whole-body samples were collected in Australia), we could not determine if there was a relationship between virome structure and diet or country. Visual inspection revealed no obvious association between termite phylogeny and virus composition and abundance, aside from members of the same species sharing similar viromes (Figure 9). Notably, however, the sampling procedure (i.e., whole-body or head-only with poly(A)+ enrichment) used to produce each library had a significant impact on virome composition, with virome abundance (*R^2^* = 0.33, *p* = 0.00865), richness (*R^2^* = 0.61, *p* = 4.27 × 10^−5^) and diversity (*R^2^* = 0.44, *p* = 0.00144) differing significantly by sampling procedure (Figure 1). Libraries prepared from whole-body samples also had a greater number of reads mapping to RNA viruses: 0.6% of total reads mapping to RNA viruses in whole-body libraries compared to 0.1% in head-only libraries.

## 4. Discussion

We present the first survey of the RNA virome of termites, demonstrating that these important organisms harbor a diverse array of RNA viruses. Overall, 50 species of termites were collected, spanning the range of the Termitoidae, yielding 20 sequencing libraries with viruses. From these data, our metatranscriptomic analysis identified 67 novel viruses related to a diverse range of RNA viruses, including the *Togaviridae, Iflaviridae, Polycipiviridae, Flaviviridae, Leviviridae, Narnaviridae, Mitoviridae, Lispivirdae, Phasmaviridae, Picobirnaviridae* and *Partitiviridae* (Figure 1 and Supplementary Table S2).

Although our sampling focused on termites, the novel RNA viruses identified likely infect a range of organisms—either the termite itself, one of its symbionts, or an ingested organism. We sought to make inferences for the hosts of all viruses discovered in this study, based on the level of virus abundance and phylogenetic positions of each virus in relation to those previously described (Figure 8 and Table 2). Although these inferences necessarily range in certainty, we suggest that the Sobemo-like, Negev-like, Picorna–Calici group, *Flaviviridae*, *Lispiviridae* and Bunya–Arena group viruses identified in this study most likely directly infect termites. Indeed, these viruses both are found in a high abundance and have relatives that are associated with arthropods [3,4,40,47,48,49,50,51].

The mitovirus-like viruses, *Picobirnaviridae* and picobirna-like novel viruses were the least abundant viruses identified, although more phylogenetically diverse. Based on their low abundance and phylogenetic context, as well as their tendency to appear in whole-body libraries, we suggest these groups most likely infect termite gut symbionts. This, in turn, provides a biological explanation for their diversity, as we would expect viruses infecting a gut microbiome to be more diverse than those infecting the termite host itself. However, the *Picobirnaviridae* are complex as they have been detected in an array of vertebrate fecal material [45,46,52], whole-body invertebrate samples [4] and soil [53]. Indeed, their ubiquity in metatranscriptomic studies may mean that they are in fact often associated with bacterial hosts. Clearly, more work is needed to determine the most likely hosts for these viruses.

An important element of this study was the high level of divergence of the viruses identified: All viruses discussed are novel, and the majority exhibit only 20% to 45% amino acid identity to their closest relatives, and this is independent of their sampling procedure or likely host. An interesting example of this is the divergent flavi-like Waxsystermes virus. This virus is highly abundant and has a genome structure typical of members of the arthropod infecting/vectored genus *Flavivirus.* It is therefore possible that Waxsystermes virus is indeed termite-infecting, although it is impossible to exclude that it is associated with a symbiont or parasite of the termite, and it is striking that Waxsystermes is most closely related to recently discovered marine-vertebrate associated viruses [54]. As such, establishing the true host of Waxsystermes virus has more broad-ranging implications for our understanding of flavivirus evolution as a whole.

The sampling of termites used in this project was not only designed to identify novel RNA viruses but to enable us to provisionally explore any relationship between these viruses and the ecology and phylogenetic background of the termites sampled. In particular, four species of termite were sampled twice, with samples representing two separate colonies per species (or the soldier and worker caste in the case of the *M. darwiniensis* samples) (Table 1). Sample replicates from the same species revealed similar viruses across all these paired samples, and it is important to note that these were the only cases in which the same virus was detected in multiple libraries. Further, this sharing of virome components was strongest in the paired soldier/worker libraries from *M. darwiniensis*: Two Partiti–Picobirna viruses, three Narna–Levi and the Luteo-Sobemo Nufsystermes virus were detected in both *M. darwiniensis* libraries 19 and 20. However, colonies sampled were collected from within the same regions, and this cross-infection of colonies may be due to geographic proximity rather than these viruses being species-specific, although the relatedness of viruses discovered from different continents may imply otherwise.

Across the samples as a whole, we detected no association between RNA virus composition or abundance and the background termite phylogeny, indicating that virome compositions lack strong heritability and can change markedly on evolutionary time scales. However, it was also notable that termite-associated representatives of a number of virus groups—Sobemo-like, *Iflaviviridae*, Mitovirus-like, Bunya–Arena, *Picobirnaviridae* and Picobirna-like viruses—clustered together on the relevant phylogenies, despite usually being collected at different locations. It may be that some virus lineages display specificity for either termites or their symbionts, as observed for a number of bacterial lineages found in termites [55]. Additional sampling of other insect and animal viromes and comparisons with those found in termites are required to assess this possibility. Strikingly, we saw a significant effect of sampling and sequencing procedure on virome characteristics: Many of our initial head-only samples yielded no virus genomes at all, and of the libraries where RNA virus genomes were identified, those from whole termite bodies had greater virus abundances and richer, more diverse RNA viromes than the head-only samples. Hence, these results show that tissue type may play a more important role in shaping virus compositions, although such a difference may also reflect the use of poly(A)+ enriched sequencing libraries in the head-only samples.

The invertebrate RNA virosphere is clearly immense. This is reflected in our identification of 67 novel, divergent viruses from the first screen of the termite RNA virome. Our work also provides insights into the types of viruses that inhabit the termite and its associated symbiont community.

## Figures and Tables

**Table 1:** Description of the termite samples used to produce the RNA sequencing libraries generated here.

Termite Species	Termite Family	Country	Diet	Procedure	Library	SRA Accession
*Basidentitermes aurivillii*	Termitidae	Kenya	Soil	Head-only	1	SRX6715944
*Amitermes* sp.	Termitidae	Australia	Wood	Head-only	2	SRX6715975
*Occasitermes* sp.1	Termitidae	Australia	Wood	Whole-body	3 ^1^	SRR8924823
*Occasitermes* sp.1	Termitidae	Australia	Wood	Whole-body	4 ^1^	SRR8924822
*Constrictotermes cavifrons*	Termitidae	French Guiana	Lichen	Head-only	5	SRX6715954
*Cephalotermes rectangularis*	Termitidae	Cameroon	Wood	Head-only	6	SRX6715969
*Labiotermes labralis*	Termitidae	French Guiana	Soil	Head-only	7	SRX6715938
*Cornitermes pugnax*	Termitidae	French Guiana	Wood	Head-only	8	SRX6715963
*Embiratermes neotenicus*	Termitidae	French Guiana	Soil	Head-only	9	SRX6715941
*Microcerotermes* sp	Termitidae	French Guiana	Wood	Head-only	10	SRX6715964
*Coptotermes acinaciformis*	Rhinotermitidae	Australia	Wood	Whole-body	11	SRR8924826
*Heterotermes ferox*	Rhinotermitidae	Australia	Wood	Whole-body	12 ^1^	SRR8924824
*Heterotermes ferox*	Rhinotermitidae	Australia	Wood	Whole-body	13 ^1^	SRR8924825
*Schedorhinotermes intermedius*	Rhinotermitidae	Australia	Wood	Whole-body	14 ^1^	SRR8924829
*Schedorhinotermes intermedius*	Rhinotermitidae	Australia	Wood	Whole-body	15 ^1^	SRR8924828
*Termitogeton planus*	Rhinotermitidae	West Papua, Indonesia	Wood	Head-only	16	SRX6715976
*Epicalotermes kempae*	Kalotermitidae	Kenya	Wood	Head-only	17	SRX6715943
*Cryptotermes secundus*	Kalotermitidae	Australia	Wood	Whole-body	18	SRR8924827
*Mastotermes darwiniensis*	Mastotermitidae	Australia	Wood	Whole-body	19 ^2^	SRR8924831
*Mastotermes darwiniensis*	Mastotermitidae	Australia	Wood	Whole-body	20 ^2^	SRR8924830

^1^ Libraries sampled from two separate colonies of the same species, ^2^ Library 19 = worker caste termites sampled, Library 20 = soldier caste termites sampled.

**Table 2:** Descriptions of each clade of novel viruses found in the termites sampled here.

Clade	Figure	Group	Novel Viruses	Host Prediction	Mean Abundance	Mean % Identity
Idaeovirus-like	Figure 5A	Hepe–Virga	1	Plant	0.0087	30.6
Negev-like	Figure 5B	Hepe–Virga	1	Termite	0.57	33.8
Sobemo-like	Figure 5C	Luteo–Sobemo	5	Termite	0.29	38.9
*Iflaviridae*	Figure 6B	Picorna–Calici	2	Termite	0.20	31.0
*Polycipiviridae*	Figure 6C	Picorna–Calici	2	Termite	0.065	38.8
Nora-like	Figure 6D	Picorna–Calici	1	Termite	0.45	29.9
Kelp fly virus related	Figure 6E	Picorna–Calici	1	Termite	0.029	24.5
*Flaviviridae*	Figure 7A	*Flaviviridae*	1	Termite	0.13	38.1
Mitovirus(-like)	Figure 7B	Narna–Levi	15	Symbiont/No inference	0.025	34.1
Narna-like	Figure 7C	Narna–Levi	1	No inference	0.14	30.0
*Leviviridae*	Figure 7D	Narna–Levi	3	Bacteria	0.0015	33.9
Lispi-like	Figure 8A	Mono–Chu	1	Termite	0.17	36.1
Bunya-like	Figure 8B	Bunya–Arena	2	Termite	0.73	39.0
*Phasmavirus*	Figure 8C	Bunya–Arena	1	Termite	0.0035	25.1
*Picobirnaviridae*	Figure 9A	Partiti–Picobirna	3	Symbiont	0.0011	46.2
Picobirna-like A	Figure 9B	Partiti–Picobirna	20	Symbiont	0.00097	32.0
Picobirna-like B	Figure 9C	Partiti–Picobirna	1	Symbiont	0.0036	27.6
Amalga-like	Figure 9D	Partiti–Picobirna	2	Symbiont	0.0030	28.3
Partiti-like A	Figure 9E	Partiti–Picobirna	1	Symbiont	0.0035	54.2
Partiti-like B	Figure 9F	Partiti–Picobirna	3	Symbiont	0.00041	53.7

## Supplementary Materials

The following are available online at https://www.mdpi.com/1999-4915/12/10/1145/s1, Figure S1: Hepe–Virga virus genome structures, Figure S2: Luteo–Sobemo virus genome structures, Figure S3: Picorna–Calici virus genome structures, Figure S4: *Flaviviridae* virus genome structures, Figure S5: Narna–Levi virus genome structures, Figure S6: Mono–Chu virus genome structures, Figure S7: Bunya–Arena virus genome structures, Figure S8: Partiti–Picobirna virus genome structures, Table S1: Termite species sampled, country of collection, SRA accessions and total numbers of reads/contigs for all libraries used in this study, Table S2: Abundance, phylogenetic and genome structure information for all viruses identified in this study, Table S3: The number of viral taxa analyzed and alignment length for multiple sequence alignments used in the phylogenetic analysis.

## References

[1] Mora C., Tittensor D.P., Adl S., Simpson A.G., Worm B. (2011). How many species are there on earth and in the ocean?. PLoS Biol..

[2] RefSeq Genome Database. https://ncbi.nlm.nih.gov/genome/viruses/.

[3] Li C.-X., Shi M., Tian J.-H., Lin X.-D., Kang Y.-J., Chen L.-J., Qin X.-C., Xu J., Holmes E.C., Zhang Y.-Z. (2015). Unprecedented genomic diversity of RNA viruses in arthropods reveals the ancestry of negative-sense RNA viruses. eLife.

[4] Shi M., Lin X.-D., Tian J.-H., Chen L.-J., Chen X., Li C.-X., Qin X.-C., Li J., Cao J.-P., Eden J.-S. (2016). Redefining the invertebrate RNA virosphere. Nature.

[5] Krishna K., Grimaldi D.A., Krishna V., Engel M.S. (2013). Treatise on the isoptera of the world: Termitidae. Bull. Am. Mus. Nat. Hist..

[6] Brune A. (2014). Symbiotic digestion of lignocellulose in termite guts. Nat. Rev. Micro..

[7] Bucek A., Šobotník J., He S., Shi M., Mcmahon D.P., Holmes E.C., Roisin Y., Lo N., Bourguignon T. (2019). Evolution of termite symbiosis informed by transcriptome–based phylogenies. Curr. Biol..

[8] Tikhe C.V., Husseneder C. (2018). Metavirome sequencing of the termite gut reveals the presence of an unexplored bacteriophage community. Front. Microbiol..

[9] Altizer S., Nunn C.L., Thrall P.H., Gittleman J.L., Antonovics J., Cunningham A.A., Dobson A.P., Ezenwa V., Jones K.E., Pedersen A.B. (2003). Social organization and parasite risk in mammals: Integrating theory and empirical studies. Annu. Rev. Ecol. Evol. Syst..

[10] Pie M.R., Rosengaus R.B., Traniello J.F. (2004). Nest architecture, activity pattern, worker density and the dynamics of disease transmission in social insects. J. Theol. Biol..

[11] Manley R., Boots M., Wilfert L. (2015). Review: Emerging viral disease risk to pollinating insects: Ecological, evolutionary and anthropogenic factors. J. Appl. Ecol..

[12] Schoonvaere K., Smagghe G., Francis F., de Graaf D.C. (2018). Study of the metatranscriptome of eight social and solitary wild bee species reveals novel viruses and bee parasites. Front. Microbiol..

[13] Beaurepaire A., Piot N., Doublet V., Antunez K., Campbell E., Chantawannakul P., Chejanovsky N., Gajda A., Heerman M., Panziera D. (2020). Diversity and global distribution of viruses of the western honey bee *Apis mellifera*. Insects.

[14] Remnant E.J., Shi M., Buchmann G., Blacquière T., Holmes E.C., Beekman M., Ashe A. (2017). A diverse range of novel RNA viruses in geographically distinct honey bee populations. J. Virol..

[15] Yanez O., Piot N., Dalmon A., de Miranda J., Chantawannakul P., Panziera D., Amiri E., Smagghe G., Schroeder D., Chejanovsky N. (2020). Bee viruses: Routes of infection in hymenoptera. Front. Microbiol..

[16] Chouvenc T., Mullins A.J., Efstathion C.A., Su N.-Y. (2013). Virus-like symptoms in a termite (*isoptera*: Kalotermitidae) field colony. Fla. Entomol..

[17] Levin D.B., Adachi D., Williams L.L., Myles T.G. (1993). Host specificity and molecular characterization of the *Entomopoxvirus* of the lesser migratory grasshopper, *Melanoplus sanguinipes*. J. Invertebr. Pathol..

[18] Al Fazairy A.A., Hassan F.A. (1988). Infection of termites by spodoptera littoralis nuclear polyhedrosis virus. Int. J. Trop. Insect Sci..

[19] Pramono A.K., Kuwahara H., Itoh T., Toyoda A., Yamada A., Hongoh Y. (2017). Discovery and complete genome sequence of a bacteriophage from an obligate intracellular symbiont of a cellulolytic protist in the termite gut. Microbes Environ..

[20] Rosario K., Mettel K.A., Benner B.E., Johnson R., Scott C., Yusseff-Vanegas S.Z., Baker C.C.M., Cassill D.L., Storer C., Varsani A. (2018). Virus discovery in all three major lineages of terrestrial arthropods highlights the diversity of single-stranded DNA viruses associated with invertebrates. PeerJ.

[21] Kerr M., Rosario K., Baker C.C.M., Breitbart M. (2018). Discovery of four novel circular single-stranded DNA viruses in fungus-farming termites. Genome Announc..

[22] Käfer S., Paraskevopoulou S., Zirkel F., Wieseke N., Donath A., Petersen M., Jones T.C., Liu S., Zhou X., Middendorf M. (2019). Re-assessing the diversity of negative strand RNA viruses in insects. PLoS Pathog..

[23] Kapelinskaya T.V., Martynova E.U., Korolev A.L., Schal C., Mukha D.V. (2008). Transcription of the German cockroach densovirus BgDNV genome: Alternative processing of viral RNAs. Dokl. Biochem. Biophys..

[24] Mukha D.V., Chumachenko A.G., Dykstra M.J., Kurtti T.J., Schal C. (2006). Characterization of a new densovirus infecting the German cockroach, *Blattella germanica*. J. Gen. Virol..

[25] Grabherr M.G., Haas B.J., Yassour M., Levin J.Z., Thompson D.A., Amit I., Adiconis X., Fan L., Raychowdhury R., Zeng Q. (2011). Full-length transcriptome assembly from RNA-Seq data without a reference genome. Nat. Biotechnol..

[26] Camacho C., Coulouris G., Avagyan V., Ma N., Papadopoulos J., Bealer K., Madden T.L. (2009). Blast+: Architecture and applications. BMC Bioinform..

[27] Marchler-Bauer A., Bryant S.H. (2004). CD-Search: Protein domain annotations on the fly. Nucleic Acids Res..

[28] Besemer J., Lomsadze A., Borodovsky M. (2001). GeneMarkS: A self-training method for prediction of gene starts in microbial genomes. Implications for finding sequence motifs in regulatory regions. Nucleic Acids Res..

[29] Li B., Dewey C.N. (2011). RSEM: Accurate transcript quantification from RNA-seq data with or without a reference genome. BMC Bioinform..

[30] Katoh K., Standley D.M. (2013). MAFFT multiple sequence alignment software version 7: Improvements in performance and usability. Mol. Biol. Evol..

[31] Capella-Gutiérrez S., Silla-Martínez J.M., Gabaldón T. (2009). TrimAl: A tool for automated alignment trimming in large-scale phylogenetic analyses. Bioinformatics.

[32] Guindon S., Gascuel O. (2003). A simple, fast, and accurate algorithm to estimate large phylogenies by maximum likelihood. Syst. Biol..

[33] Paradis E., Schliep K. (2018). ape 5.0: An environment for modern phylogenetics and evolutionary analyses in R. Bioinformatics.

[34] Yu G., Lam T.T., Zhu H., Guan Y. (2018). Two methods for mapping and visualizing associated data on phylogeny using ggtree. Mol. Biol. Evol..

[35] Revell L.J. (2012). Phytools: An R package for phylogenetic comparative biology (and other things). Methods Ecol. Evol..

[36] Oksanen J., Blanchet F.G., Friendly M., Kindt R., Legendre P., McGlinn D., Minchin P.R., O’Hara R.B., Simpson G.L., Solymos P. (2019). Vegan: Community Ecology Package. https://CRAN.R-project.org/package=vegan.

[37] McMurdie P.J., Holmes S. (2013). Phyloseq: An R package for reproducible interactive analysis and graphics of microbiome census data. PLoS ONE.

[38] Hothorn T., Bretz F., Westfall P. (2008). Simultaneous inference in general parametric models. Biom. J..

[39] Wickham H. (2016). Ggplot2: Elegant Graphics for Data Analysis.

[40] Harvey E., Rose K., Eden J.-S., Lo N., Abeyasuriya T., Shi M., Doggett S.L., Holmes E.C. (2019). Extensive diversity of RNA viruses in australian ticks. J. Virol..

[41] Olendraite I., Brown K., Valles S.M., Firth A.E., Chen Y., Guérin D.M., Hashimoto Y., Herrero S., de Miranda J.R., Ryabov E. (2019). ICTV virus taxonomy profile: *Polycipiviridae*. J. Gen. Virol..

[42] Shackelton L.A., Holmes E.C. (2008). The role of alternative genetic codes in viral evolution and emergence. J. Theor. Biol..

[43] Nibert M.L., Vong M., Fugate K.K., Debat H.J. (2018). Evidence for contemporary plant mitoviruses. Virology.

[44] Maes P., Adkins S., Alkhovsky S.V., Avšič-Županc T., Ballinger M.J., Bente D.A., Beer M., Bergeron É., Blair C.D., Briese T. (2019). Taxonomy of the order bunyavirales: Second update 2018. Arch. Virol..

[45] Yinda C.K., Vanhulle E., Conceição-Neto N., Beller L., Deboutte W., Shi C., Ghogomu S.M., Maes P., Van Ranst M., Matthijnssens J. (2019). Gut virome analysis of cameroonians reveals high diversity of enteric viruses, including potential interspecies transmitted viruses. mSphere.

[46] Krishnamurthy S.R., Wang D. (2018). Extensive conservation of prokaryotic ribosomal binding sites in known and novel picobirnaviruses. Virology.

[47] Viljakainen L., Borshagovski A.M., Saarenpää S., Kaitala A., Jurvansuu J. (2020). Identification and characterisation of common glow-worm RNA viruses. Virus Genes.

[48] Harvey E., Rose K., Eden J.-S., Lawrence A., Doggett S.L., Holmes E.C. (2019). Identification of diverse arthropod associated viruses in native australian fleas. Virology.

[49] Debat H.J. (2017). An RNA virome associated to the golden orb-weaver spider *Nephila clavipes*. Front. Microbiol..

[50] Liu Y., Shen D., Zhou F., Wang G., An C. (2014). Identification of immunity-related genes in *Ostrinia furnacalis* against entomopathogenic fungi by RNA-seq analysis. PLoS ONE.

[51] Carter J.M., Baker S.C., Pink R., Carter D.R., Collins A., Tomlin J., Gibbs M., Breuker C.J. (2013). Unscrambling butterfly oogenesis. BMC Genom..

[52] Martínez L.C., Masachessi G., Carruyo G., Ferreyra L.J., Barril P.A., Isa M.B., Giordano M.O., Ludert J.E., Nates S.V. (2010). Picobirnavirus causes persistent infection in pigs. Infect. Genet. Evol..

[53] Starr E.P., Nuccio E.E., Pett-Ridge J., Banfield J.F., Firestone M.K. (2019). Metatranscriptomic reconstruction reveals RNA viruses with the potential to shape carbon cycling in soil. Proc. Natl. Acad. Sci. USA.

[54] Parry R., Asgari S. (2019). Discovery of novel crustacean and cephalopod flaviviruses: Insights into the evolution and circulation of flaviviruses between marine invertebrate and vertebrate Hosts. J. Virol..

[55] Bourguignon T., Lo N., Dietrich C., Šobotník J., Sidek S., Roisin Y., Brune A., Evans T.A. (2018). Rampant host switching shaped the termite gut microbiome. Curr. Biol..

